# [Corrigendum] CDKN2A (p16INK4A) affects the anti-tumor effect of CDK inhibitor in somatotroph adenomas

**DOI:** 10.3892/ijmm.2026.5732

**Published:** 2026-01-14

**Authors:** Yiyuan Chen, Zhenye Li, Qiuyue Fang, Hongyun Wang, Chuzhong Li, Hua Gao, Yazhuo Zhang

Int J Mol Med 47: 500-510, 2021; DOI: 10.3892/ijmm.2020.4807

Following the publication of the above article and an expression of concern statement (doi: 10.3892/ijmm.2025.5680) after it had been drawn to the Editor's attention by an interested reader that, regarding the western blot data shown in Fig. 5 on p. 507, the first set of GAPDH bands for the GH3 cell line were strikingly similar to the EGFR protein bands shown for the GT1-1 cell line in the adjacent set of gels, the authors have now replied to the Editorial Office to explain the apparently anomalous appearance of this figure.

After having examined their original data, the authors have realized that this figure was assembled incorrectly; essentially, the wrong data were included in this figure to portray the GAPDH bands for the GH3 cell line. The revised version of Fig. 5, now showing the correct GAPDH data for the GH3 cell line, is featured on the next page. The authors can confirm that the error made during the assembly of Fig. 5 did not have a significant impact on either the results or the conclusions reported in this study, and all the authors agree with the publication of this Corrigendum. The authors are grateful to the Editor of *International Journal of Molecular Medicine* for allowing them the opportunity to publish this Corrigendum; furthermore, they apologize to the readership of the Journal for any inconvenience caused.

## Figures and Tables

**Figure 5:**
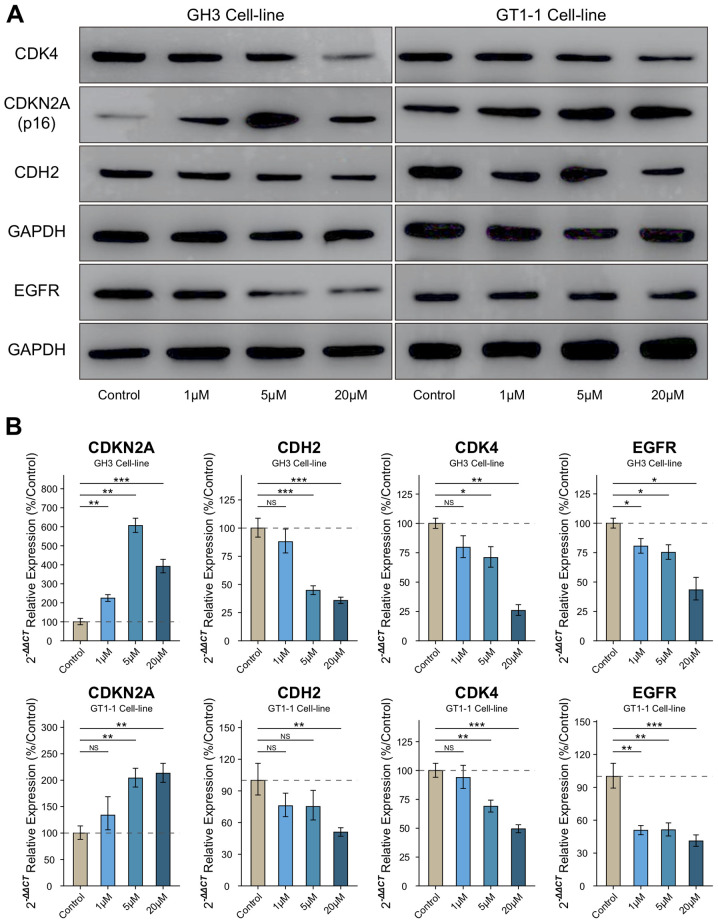
Palbociclib relieved the levels of CDH2, CDK4 and EGFR accompanied by upregulation of CDKN2A in various concentrations (1, 5, and 20 *μ*M). (A) Bands of western blot assay. (B) Statistical analysis of mRNA level in RT-qPCR assay. compared to control group. ^*^P<0.05, ^**^P<0.01, ^***^P<0.001, n=3.

